# Functionalized Silicon Electrodes Toward Electrostatic Catalysis

**DOI:** 10.3389/fchem.2021.715647

**Published:** 2021-07-27

**Authors:** Long Zhang, Xiaohua Yang, Shun Li, JianMing Zhang

**Affiliations:** ^1^ Institute of Quantum and Sustainable Technology (IQST), School of Chemistry and Chemical Engineering, Jiangsu University, Zhenjiang, China; ^2^ Foshan (Southern China) Institute for New Materials, Foshan, China

**Keywords:** electrostatic catalysis, semiconductor silicon, space charge, electric double layer, alkoxyamine

## Abstract

Oriented external electric fields are now emerging as “smart effectors” of chemical changes. The key challenges in experimentally studying electrostatic catalysis are (i) controlling the orientation of fields along the reaction axis and (ii) finely adjusting the magnitudes of electrostatic stimuli. Surface models provide a versatile platform for addressing the direction of electric fields with respect to reactants and balancing the trade-off between the solubility of charged species and the intensity of electric fields. In this mini-review, we present the recent advances that have been investigated of the electrostatic effect on the chemical reaction on the monolayer-functionalized silicon surfaces. We mainly focus on elucidating the mediator/catalysis role of static electric fields induced from either solid/liquid electric double layers at electrode/electrolyte interfaces or space charges in the semiconductors, indicating the electrostatic aspects is of great significance in the semiconductor electrochemistry, redox electroactivity, and chemical bonding. Herein, the functionalization of silicon surfaces allows scientists to explore electrostatic catalysis from nanoscale to mesoscale; most importantly, it provides glimpses of the wide-ranging potentials of oriented electric fields for switching on/off the macroscale synthetic organic electrochemistry and living radical polymerization.

## Introduction

At present, increasing attention has been paid to use oriented electric fields as “smart reagents” to catalyze non-electrochemical reactions, namely, electrostatic catalysis (Shaik et al., 2016; Ciampi et al., 2018). The concept is that a controllable electric field enables chemists to control catalysis/inhibition (Meir et al., 2010) of chemical/biological reactions at will and hence establishes a new approach to future chemistry and biology, from the adjustment of enzyme activity in biological processes (Warshel et al., 2006; Fried et al., 2014) to the manipulation of barrier heights of chemical reactions (Zhang et al., 2018a; Wang et al., 2019) or functions of molecular devices (Foroutan-Nejad et al., 2016; Jaroš et al., 2019). However, electrostatic catalysis application is still in its infancy in experimental investigations but massively applied in theoretical predictions. This is because the intrinsic feature of an electric field as a vector suffers from limitations in adjusting the orientation of an electric field against the reaction axis and gauging the precise strength of the electric field applied on the reactions. Furthermore, electrostatic effects are predominant in the gas phase and progressively attenuate with an increasing polarity of media, whereas the solubility of charged residues is poor in non- or less-polar solvents (Gryn'ova et al., 2013; Gryn’ova and Coote, 2013). Therefore, one needs to address two challenges for extending electrostatic catalysis to practical reactions: (i) controlling the direction and strength of an external electric field at will and (ii) balancing the trade-off between the solubility of charged species and the magnitude of electric fields.

It was not until 2016 that Coote et al. provided a proof of concept that an electric field could accelerate a carbon–carbon bond-forming reaction through scanning tunneling microscope-break junction (STM-BJ) experiments (Aragonès et al., 2016); this nanoscale technique was subsequently selected as a versatile platform to trigger some chemical changes by controlling the orientation and intensity of electric fields deliberately (Xiang and Tao, 2016; Zang et al., 2019). Although this offered the possibility of using easily accessible model systems to explore electrostatic catalysis phenomena experimentally, the proposed STM-BJ surface system is not on a practical scale, and only limited molecules/specific reactions are employed to match this experimental setup; for example, some single-molecule junctions are constructed based on gold–carbon bond formation (Hong et al., 2012; Starr et al., 2020). To realize electrostatic catalysis beyond individual molecules, one must be able to devise monolayer surface systems by coupling interested molecules to the sturdiness of a solid device (Chia et al., 2001; Gooding and Ciampi, 2011). The semiconductor silicon electrode, technologically the most relevant electrode material of our age (Ball, 2005; Ciampi et al., 2010), is still promoting the rapid development of electrochemistry (Choudhury et al., 2015; Vogel et al., 2017), molecular electronics (Rakshit et al., 2004; Vilan and Cahen, 2017), quantum computing (Hill et al., 2015; Tosi et al., 2017), and spintronics (Flatte, 2009; Jansen, 2012). The major reasons for selecting a silicon electrode for the electrostatic catalysis study are as follows: (i) well-developed approaches are available for preparing monolayers on silicon electrodes; (ii) it has excellent photoelectrochemical properties in the electrode–electrolyte interface (Rajeshwar, 2007); and (iii) it is easily accessible for surface-sensitive X-ray spectroscopic characterizations with the Si/C contrast (Ciampi et al., 2009). These advantages have made the silicon electrode an ideal platform for investigating electrostatic catalysis in mesoscale processes. The functionalization of silicon substrates and semiconductor electrochemistry will be briefly introduced below for readers to appreciate how the electrostatic effect works on monolayer films. Specifically, we present a detailed summary on the advances of electrostatic catalysis of redox and non-redox reactions on functionalized silicon electrodes, suggesting the electric fields can be used as “smart reagents” for regulating organic surface synthesis and switching ON/OFF the nitroxide-mediated polymerization.

## Assembly of Organic Monolayers on Silicon Surfaces

Silicon surface models provide us with an efficient strategy to address the orientation of external electric fields with respect to reactants as well as balance the discrepancy between the solubility of charged species and the magnitude of external electric fields. Our focus is first dedicated to the preparation of stable silicon monolayer films, minimizing/avoiding the formation of silicon oxides during measurements and ensuring the catalytic effects of electric fields. When it comes to preparation of chemically well-defined organic monolayers on silicon electrodes, numerous protocols are available to prepare Si-C, Si-O, and Si-N bound layers (Ciampi et al., 2010; Gonçales et al., 2020). Specifically, Si-C bound surface modifications are one of the most common covalent attachments on a silicon electrode. To obtain the robust Si-C linked monolayers and hence exact electron-transport studies, the insulating silicon oxide layers on silicon substrates typically need to be passivated by chemical etching in either fluoride-containing solution (Chabal et al., 1989; Niwano et al., 1992) or alkaline aqueous (Zhang, 2007; Clark et al., 2010), leading to the formation of hydrogen-terminated (Si-H) silicon surfaces. The resulting Si-H surfaces are unequivocally attractive to the attachment of molecular monolayers by wet chemical routes. This is because the Si-H functionality possesses some intriguing properties; for instance, it can be prepared easily (Higashi et al., 1990), has short-term tolerance to atmosphere and aqueous media (Linford et al., 1995), and shows inert reactivity toward common organic media (Bateman et al., 1998). Two methods widely used in the prevent of silicon oxide formation were introduced in this review: (1) insertion of an unsaturated molecule (i.e., alkenes and alkynes) into Si-H bonds, which is known as hydrosilylation (Ciampi et al., 2010), and (2) formation of a Si-C linkage on non-oxidized Si-H surfaces, which can be carried out by thermal-triggered hydrosilylation under appropriate conditions (Linford and Chidsey, 1993; Linford et al., 1995). In contrast, photochemical irradiation can also promote hydrosilylation of unsaturated compounds *via* homolytic cleavage of Si-H bonds at room temperature (Terry et al., 1997; Stewart and Buriak, 1998). The relevant procedures will be detailed in the below sections.

## Non-Ideal Voltammograms in Semiconductor Electrochemistry

**FIGURE 1:**
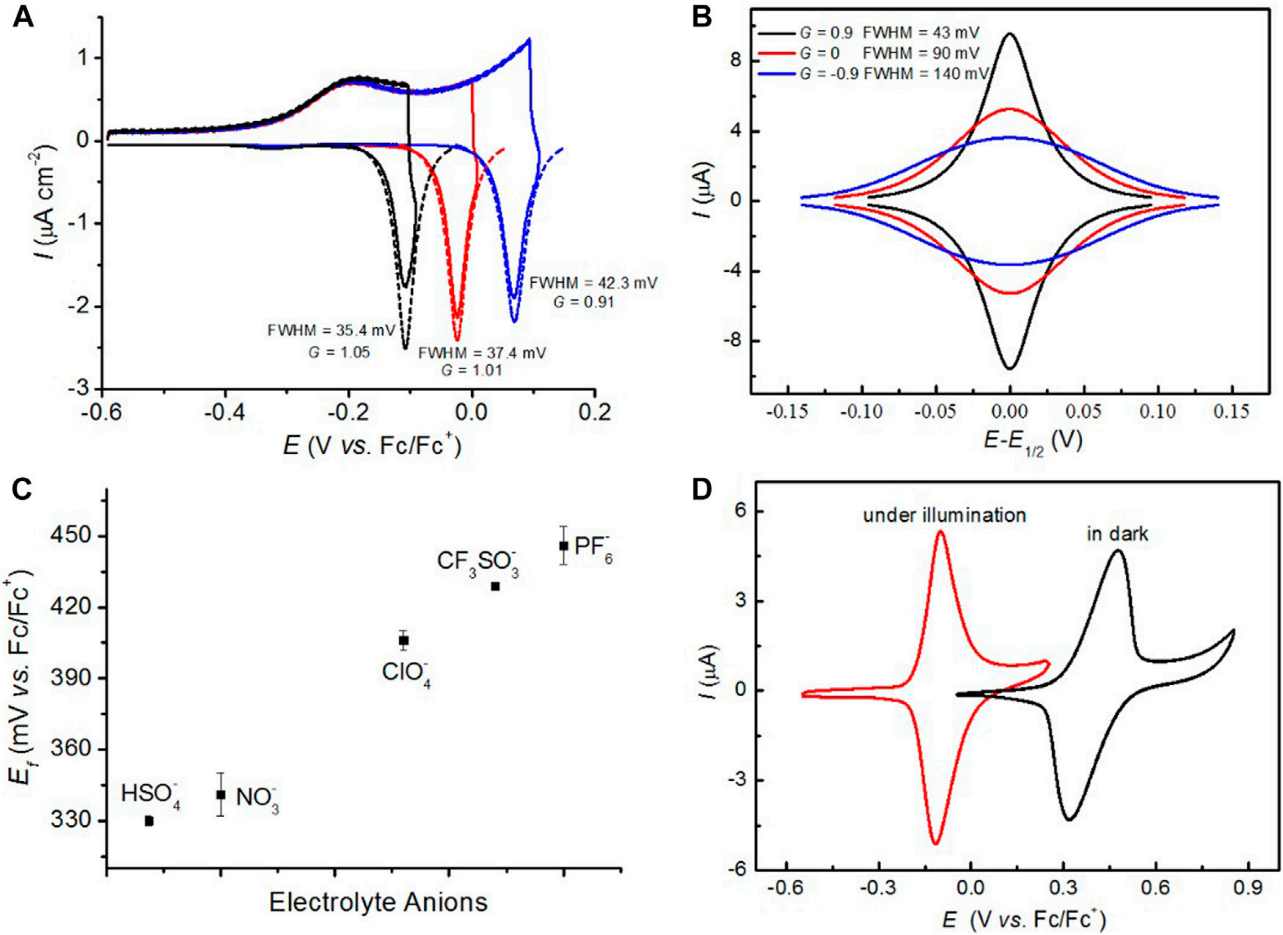
**(A)** A non-ideal “peak inversion” voltammogram is induced on the TEMPO monolayer modified poorly doped Si(100) electrode when the illumination is switched off at the anodic vertex (0.4, 0.5, or 0.6 V) in 0.1 M dichloromethane/tetrabutylammonium perchlorate (DCM/TBAClO_4_). The simulated CVs (symbol), calculated FWHM values, and refined self-interaction parameter *G* are also indicated. **(B)** Simulated CVs for a reversible one-electron redox couple where both species are strongly adsorbed on the electrode surface. The Frumkin “*G*” term that leads to the isotherm under equilibrium is varied between −0.9 and 0.9, suggesting the extent of the electrostatic interactions can be parametrized by simply introducing changes to the Frumkin *G* factor. The electrode area is set to 0.28 cm^2^, surface coverage is 2 × 10^−10^ mol cm^−2^, and the voltage sweep rate is 100 mV s^−1^. **(C)** Shifts of the experimental $E_{f}^{0}\;$as a function of the Lewis basicity of the electrolyte anions. The experimental values of $E_{f}^{0}$ are obtained from the CV data of the TEMPO monolayer in acetonitrile solution containing different tetrabutylammonium salts. The indicated error bars were calculated based on 95% confidence limit of the mean with six repetitive measurements for each electrolytic system. **(D)** CVs for TEMPO prepared on lowly doped Si(100) electrodes: redox waves in dark (black line) and their underpotential shifts under illumination (red line).

Scientists occasionally encountered non-ideal voltammograms [anti-thermodynamic inverted redox peaks and <90.6 mV full width at half maximum (FWHM) value] while dealing with redox reactions that occur in semiconductor electrodes (Bolts and Wrighton, 1979; Zhang et al., 2016). The current signal obtained from an electrified interface was always treated as an indicative of an overall rate; hence, these non-idealities were immediately disregarded as electrochemical flaws or artifacts. We recently found that these non-ideal electrochemical phenomena can be reproduced and are not flawed data; they are the manifestations of an electrostatic interplay between surface charged species and space charges in the semiconductors. Zhang et al. studied the nitroxide’s electrochemical behavior in solvents of different dielectric constants by tethering 2,2,6,6-tetramethyl-1-piperidinyloxy (TEMPO) monolayers to the lowly doped Si(100) electrode (Zhang et al., 2016). The cyclic voltammograms (CVs) with only sweeps toward the anodic direction were recorded under illumination, and the light source was immediately switched off at the anodic vertex. For example, a normal oxidized wave is usually observed under illumination as shown in Figure 1A, but a very sharp reduced peak appeared instantly while switching off the light, which is tentatively ascribed to formation of a space-charge barrier for electrons to enter the semiconductor, hence affecting the electronic band of silicon electrode and bending extent of conduction/valence bands by surface positively charged species (Zhang et al., 2016; Vogel et al., 2017). Therefore, a reduction peak appeared at a potential which is more anodic than oxidative, which is the result of an electrostatic effect between oxoammonium (oxide of nitroxide) and semiconductor space charge. Subsequently, we found that the FWHM is largely dependent on the surface coverage of electroactive units; that is, its values drop from 142 to 55–75 mV at ferrocene-modified poorly doped Si(111) electrodes when ferrocene’s densities change from 2.9 × 10^−10^ to 1.7 × 10^–10^ mol cm^−2^, respectively (Vogel et al., 2017). These consequences are attributed to the fact that the dominating electrostatic forces are converted from repulsive interactions between ferrocenes (142 mV FWHM) to attractive interactions between ferrocenium and space charges (55–75 mV FWHM). As can be seen from Figure 1B, the outcomes can be further verified by the fitted Frumkin interaction parameter (*G*), describing that the extent of attractive and repulsive interactions between molecules (Laviron, 1974) is directly related to the FWHM values. *G* is defined zero when there is no interaction involving electroactive redox probes. The band-bending degree in the depleted semiconductor space charge is greatly relied on the external stimuli; for example, the band would bend more while the external illumination intensity is increasing (Zhang et al., 2016) or the surface-confined redox probe (Vogel et al., 2017) is decreasing, suggesting that the electrostatic interaction on the surface-tethered species is in favor of attractive forces, with the *G* value increasing above zero. On top of this, the effect of space charge on the electrostatic interaction becomes more evident with the decrease of the solvent’s dielectric constant. These findings provide us with immediate implications for a comprehensive understanding of kinetic and thermodynamic analyses of charge transport at the electrified semiconductor/liquid interface (Vogel et al., 2019), as well as enable one to get more insight into electrostatic effects on chemical reactivity (Zhang et al., 2018a).

## The Electrostatic Effect on Redox Electroactivity

Persistent organic free radicals have been extensively studied in chemistry and biology (Fischer, 2001), and TEMPO and nitroxides have attracted interests in the study of electrostatic catalysis as theoretical models (Gryn'ova et al., 2013; Gryn’ova and Coote, 2013). In spite of the reversible and stable one-electron electrochemical process of TEMPO oxidation in both aqueous and organic media, experimental works investigating the electrochemical behavior of nitroxide monolayers are rare, which is ascribed to the difficulty in preserving the open-shell state of radicals during the modification procedures (Niermann et al., 2006). Ciampi’s group established a two-step procedure to prepare TEMPO monolayers on Si(100) electrodes and protect their unpaired electrons (Zhang et al., 2016). Clean Si(100) wafers are first etched with degassed 40% aqueous ammonium fluoride to generate hydrogen-terminated Si(100) [Si(100)-H], followed by the functionalization of Si(100)-H *via* thermal hydrosilylation of 1,8-nonadiyne to produce alkyne-terminated monolayers at 160°C (Ciampi et al., 2007; Ciampi et al., 2009). The final TEMPO monolayers are obtained by Cu(I)-catalyzed alkyne-azide cycloaddition reactions (Meldal and Tornøe, 2008) between acetylene-functionalized Si(100) surfaces and azido group-functionalized TEMPO molecules. This surface system is used to explore the effect of electrostatic interactions between grafted TEMPO moieties and electrolyte anions or space charge in the semiconductor on the redox electroactivity of nitroxide monolayers. When TEMPO molecules are tethered to a highly doped silicon electrode, the apparent formal potential $E_{f}^{0}$ of a surface nitroxide/oxoammonium redox couple is closely related to electrolyte anions, and it moves progressively anodically in the sequence HSO_4_^−^ (330 ± 2 mV) < NO_3_^−^ (341 ± 9 mV) < ClO_4_^−^ (406 ± 4 mV) < CF_3_SO_3_^−^ (429 ± 1 mV) < PF_6_^−^ (446 ± 8 mV). With the exception of HSO_4_^−^, the magnitude of the displacement in $E_{f}^{0}$ shifts positively gradually with the decrease of the anions’ Lewis basicity (Figure 1C). The stronger than expected basicity of HSO_4_^−^ compared with the other anions can be rationalized by the formation of a stabilizing H-bond with the 1,2,3-triazole ring. The displacement in $E_{f}^{0}$ can be interpreted by the Lewis acid–base reaction (*i.e.*, oxoammonium–electrolyte anion interaction), and this force leads to a reduction in the thermodynamic cost for oxidizing TEMPO. Consequently, the electrochemical activity of TEMPO monolayers can be predictably manipulated by simply changing the electrolytes. Conversely, for immobilizing nitroxide radicals on the poorly doped Si(100) electrode, there is an internal electric field induced by the semiconductor space charge, which can be applied to drive the redox reaction of TEMPO contrathermodynamically. To our best knowledge, the kinetic barrier of depleted dark semiconductor electrodes can be effectively controlled, or even removed, upon illumination with different energies; in particular, the formed internal electric field in the space-charge region of semiconductors can predictably tune the electroactivity of redox species attached to the electrode surface, hence implying the electrostatic aspect in the semiconductor electrochemistry. Figure 1D reveals the effect of electrostatic interactions between the tethered oxoammonium and the semiconductor space charge on the kinetic and thermodynamic parameters of surface TEMPO redox reactions; for instance, compared to the faradaic response in the dark, the illuminated current–voltage curves of TEMPO electrochemical reactions present a 1500-fold higher electron transfer kinetics as well as around 500 mV more negative $E_{f}^{0}$ and decreased FWHM values. Furthermore, the extent of electrostatic effect within the solid/liquid electric double layer and the semiconductor space charge is possibly measured by characterizing the changes in the voltammetric response.

## Electrostatic Catalysis of Alkoxyamine Cleavage

**FIGURE 2:**
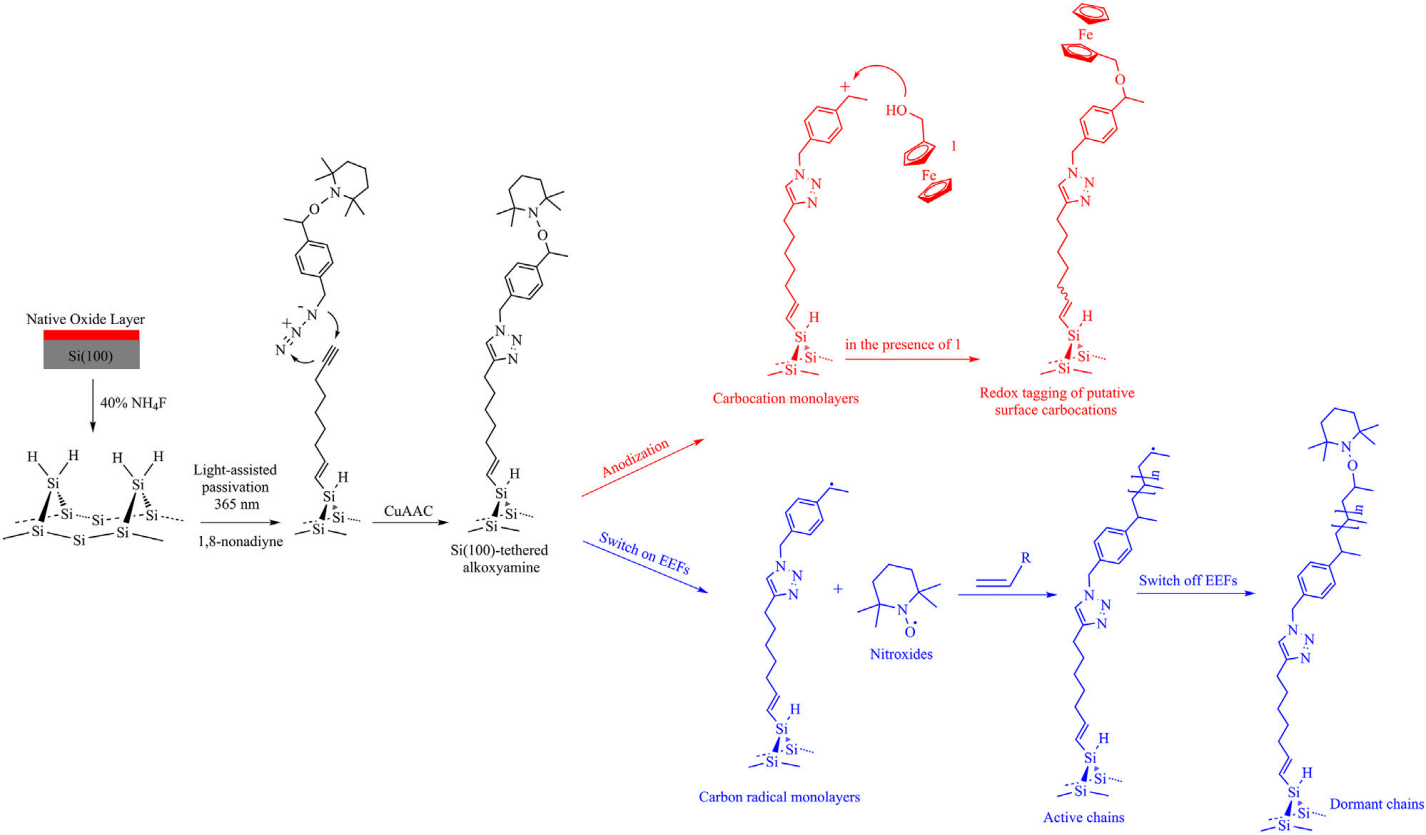
Light-assisted (365 nm) hydrosilylation of 1,8-nonadiyne to passivate a Si(100)-H surface and covalent attachment of alkoxyamines *via* “click” reactions to yield an alkoxyamine monolayer. (Red route) Anodization of the alkoxyamine monolayer in the presence of the nucleophile ferrocenemethanol leads to release of TEMPO diffused in the electrolyte and formation of a redox-tagged monolayer by the reaction between ferrocenemethanol and the putative surface-tethered carbocation monolayer. (Blue route) External electric fields (EEFs) triggered decomposition of alkoxyamines to generate active carbon-centered radical monolayers for initiating the living radical polymerization. The polymerized process can be terminated by recombining the diffusive TEMPO while switching off EEFs.

Beyond utilizing electric fields to catalyze redox (Zhang et al., 2016) or single-molecule (Peiris et al., 2019) reactions on silicon substrates, chemists are devoted to the goal of catalyzing non-redox reactions by applying an external electric field. Alkoxyamines are arguably the prime precursors in the field of nitroxide-mediated polymerization, design of smart materials, and theranostics (Audran et al., 2014). However, they require either relatively high temperatures or high-energy irradiation to trigger their homolytic process with either unfortunate consequences of unwanted side reactions or carcinogenesis of living cells, respectively. Electrochemically controllable C-ON cleavage is recently reported using electricity as an alternative stimulus of alkoxyamines’ decomposition at room temperature (Zhang et al., 2018a), indicating that the electrochemical cleavage of alkoxyamines is sensitive to electrostatic environments such as molecular structure and the supporting electrolytes and solvents (Hammill et al., 2019; Noble et al., 2019). The magnitude of the electrostatic effect on the homolytic cleavage of alkoxyamines is quantified by the formation of an alkoxyamine molecular junction in the STM-BJ setup. In contrast, it can also undergo a one-electron electrochemical oxidation to form an unstable cation radical intermediate, which rapidly fragments into a nitroxide and a carbocation, implying a sequential electrochemical–chemical–electrochemical (ECE) process. With the aid of digital simulations and quantum computations, a series of voltammetric experiments indicate that the collapse of the alkoxyamine cation radicals is largely influenced by electrostatic environments (*i.e.*, solvents, ion-pairing, and electrolytic concentration) in the electrolytes. Importantly, when the alkoxyamines are grafted on the silicon surface, one can control the number of fragments by changing the anodization time of applied positive biases, suggesting a versatile approach for generating *in situ* active nitroxides and carbocations used to regulate chemical synthesis. For example, we have covalently attached an alkoxyamine derivative to a Si(100) surface and applied an anodic bias to trigger its decomposition into a diffusive nitroxide radical and a surface-tethered carbocation (Zhang et al., 2018b). Different from the abovementioned thermal-induced hydrosilylation, acetylanylated Si(100) was prepared by reacting Si(100)-H with 1,8-nonadiyne by UV light-assisted hydrosilylation under an argon atmosphere. Subsequently, the synthesized alkoxyamines were attached to alkyne-functionalized Si(100) *via* a Cu(I)-catalyzed “click reaction”; as can be seen from Figure 2 (red path), a stable alkoxyamine monolayer can be switched into a highly reactive carbocation surface under a positive bias stimuli and the *in situ* electrogenerated surface-confined carbocations immediately trap the preexisting nucleophiles (*i.e.*, ferrocenemethanol), which is characterized by using the cyclic voltammogram with an obvious ferrocene redox signature. By changing the anodization time applied upon alkoxyamine monolayers, one is able to control the density of active carbocations and hence the coverage of a formed redox probe tagged monolayer. This proof-of-concept experiment expands the electrostatic effect to the regulation of organic electrosynthesis (Norcott et al., 2019).

## Conclusions and Perspectives

Since the first experimental evidence about the electrostatic catalysis of the C–C bond formation at individual-molecule interfaces (Aragonès et al., 2016), using oriented electric fields as smart catalysts to catalyze chemical synthesis provides scientists with a novel idea to revolutionize conventional reactions catalyzed by specific catalysts (Shaik et al., 2016; Ciampi et al., 2018). Although the development of electrostatic catalysis has already attracted wide attention, research using electrostatics as an effector of chemical changes is still in its infancy. Most work on electrostatic effects so far has been limited to theoretical investigations and individual-molecule studies because of the orientation and strength issues; therefore, the development of versatile platforms to control the orientation and intensity of an external electric field at will is an extremely attractive approach toward electrostatic catalysis experiments. Functionalization of the semiconductor silicon surface takes the developing trajectory of the electrostatic effect from controlling chemical bonding study at the nanoscale to manipulating chemical reactions at the mesoscale. In this review, we have summarized the progress of electrostatic catalysis based on a monolayer-functionalized silicon surface, and the contents include 1) developing the wet chemistry methods for preparing stable monolayers on silicon electrodes; 2) revealing the effect induced by space charge on the non-ideal voltammetric behaviors in the semiconductor electrochemistry; 3) studying the effect of electrostatic interactions on the electrochemical reactivity of a redox nitroxide monolayer; and 4) finding the stability of the C-ON bond in alkoxyamine being affected by the electrostatic environments in electrolytes. These experiments are, however, unlikely to be of practical value toward chemical reactions at preparative scale (Klinska et al., 2015; Zhang et al., 2020).

The prospects of electrostatic catalysis is to establish the static electric field as a “smart reagent”—a catalyst or inhibitor (Meir et al., 2010) in a broad range of chemical reactions; this will bring the concept of electrostatic catalysis into the realm of chemical methods that are both clean and green and able to process workable quantities of materials. With the knowledge that the electroactivity of nitroxide radicals can be tuned by electrostatic forces, it may aid the development of electrocatalytic heterogeneous systems coupled to the homogeneous reactions of surface-confined monolayers (Zhang et al., 2016; Che et al., 2018). For example, the heterogeneous oxidation potential of bromide to bromine is driven contrathermodynamically by up to 500 mV under illumination while tethering the redox mediator of TEMPO monolayers to poorly doped silicon electrodes compared with that of grafting TEMPO molecules on highly doped silicon surfaces. The development of polymerization strategies is also of great current significance in both fundamental studies and industrial applications of surface polymers. Electrostatically controllable C-ON homolysis of alkoxyamine molecules present a new perspective for future surface grafting methodology controlled by the electric fields in the absence of initiators, stabilizers, and dispersants, which is much less hazardous than the traditional thermal- and irradiation-triggered polymerizations. Specifically, the blue path in Figure 2 illustrates that one is able to prepare an alkoxyamine monolayer on a silicon surface and dissociate it to tethered carbon-centered radicals and free nitroxides diffused in an electrolyte bulk when an external bias is switched on. The generated carbon radical will initiate living polymerization when there are unsaturated monomers, and propagation will be stopped by recombining nitroxides to form a tethered alkoxyamine when the field is switched off. Overall, all of these findings allow expanding our understanding of electrostatic forces over chemical reactivity (Shaik et al., 2018) and enable one to design a smart electrostatic switch to govern chemical changes deliberatively.

## References

[B1] Aragonès A. C. Haworth N. L. Darwish N. Ciampi S. Bloomfield N. J. Wallace G. G. (2016). Electrostatic Catalysis of a Diels-Alder Reaction. Nature 531, 88–91. 10.1038/nature16989 26935697

[B2] Audran G. Brémond P. Marque S. R. A. (2014). Labile Alkoxyamines: Past, Present, and Future. Chem. Commun. 50, 7921–7928. 10.1039/c4cc01364f 24817073

[B3] Ball P. (2005). Silicon Still Supreme. Nat. Mater 4, 119. 10.1038/nmat1330 15689949

[B4] Bateman J. E. Eagling R. D. Worrall D. R. Horrocks B. R. Houlton A. (1998). Alkylation of Porous Silicon by Direct Reaction with Alkenes and Alkynes. Angew. Chem. Int. Edition 37, 2683–2685. 10.1002/(sici)1521-3773(19981016)37:19<2683::aid-anie2683>3.0.co;2-y 29711624

[B5] Bolts J. M. Wrighton M. S. (1979). Chemically Derivatized N-type Semiconducting Gallium Arsenide Photoelectrodes. Thermodynamically Uphill Oxidation of Surface-Attached Ferrocene Centers. J. Am. Chem. Soc. 101, 6179–6184. 10.1021/ja00515a005

[B6] Chabal Y. J. Higashi G. S. Raghavachari K. Burrows V. A. (1989). Infrared Spectroscopy of Si(111) and Si(100) Surfaces after HF Treatment: Hydrogen Termination and Surface Morphology. J. Vacuum Sci. Tech. A: Vacuum, Surf. Films 7, 2104–2109. 10.1116/1.575980

[B7] Che F. Gray J. T. Ha S. Kruse N. Scott S. L. McEwen J.-S. (2018). Elucidating the Roles of Electric fields in Catalysis: A Perspective. ACS Catal. 8, 5153–5174. 10.1021/acscatal.7b02899

[B8] Chia S. Cao J. Stoddart J. F. Zink J. I. (2001). Working Supramolecular Machines Trapped in Glass and Mounted on a Film Surface. Angew. Chem. Int. Ed. 40, 2447–2451. 10.1002/1521-3773(20010702)40:13<2447::aid-anie2447>3.0.co;2-p 29712269

[B9] Choudhury M. H. Ciampi S. Yang Y. Tavallaie R. Zhu Y. Zarei L. (2015). Connecting Electrodes with Light: One Wire, many Electrodes. Chem. Sci. 6, 6769–6776. 10.1039/c5sc03011k 28757968 PMC5508692

[B10] Ciampi S. Böcking T. Kilian K. A. James M. Harper J. B. Gooding J. J. (2007). Functionalization of Acetylene-Terminated Monolayers on Si(100) Surfaces: a Click Chemistry Approach. Langmuir 23, 9320–9329. 10.1021/la701035g 17655337

[B11] Ciampi S. Darwish N. Aitken H. M. Díez-Pérez I. Coote M. L. (2018). Harnessing Electrostatic Catalysis in Single Molecule, Electrochemical and Chemical Systems: a Rapidly Growing Experimental Tool Box. Chem. Soc. Rev. 47, 5146–5164. 10.1039/c8cs00352a 29947390

[B12] Ciampi S. Eggers P. K. Le Saux G. James M. Harper J. B. Gooding J. J. (2009). Silicon (100) Electrodes Resistant to Oxidation in Aqueous Solutions: An Unexpected Benefit of Surface Acetylene Moieties. Langmuir 25, 2530–2539. 10.1021/la803710d 19159188

[B13] Ciampi S. Harper J. B. Gooding J. J. (2010). Wet Chemical Routes to the Assembly of Organic Monolayers on Silicon Surfaces *via* the Formation of Si-C Bonds: Surface Preparation, Passivation and Functionalization. Chem. Soc. Rev. 39, 2158–2183. 10.1039/b923890p 20393648

[B14] Clark I. T. Aldinger B. S. Gupta A. Hines M. A. (2010). Aqueous Etching Produces Si(100) Surfaces of Near-Atomic Flatness: Strain Minimization Does Not Predict Surface Morphology. J. Phys. Chem. C 114, 423–428. 10.1021/jp908527e

[B15] Fischer H. (2001). The Persistent Radical Effect: a Principle for Selective Radical Reactions and Living Radical Polymerizations. Chem. Rev. 101, 3581–3610. 10.1021/cr990124y 11740916

[B16] Flatté M. E. (2009). Silicon Spintronics Warms up. Nature 462, 419–420. 10.1038/462419a 19940906

[B17] Foroutan-Nejad C. Andrushchenko V. Straka M. (2016). Dipolar Molecules inside C70: an Electric Field-Driven Room-Temperature Single-Molecule Switch. Phys. Chem. Chem. Phys. 18, 32673–32677. 10.1039/c6cp06986j 27892557

[B18] Fried S. D. Bagchi S. Boxer S. G. (2014). Extreme Electric fields Power Catalysis in the Active Site of Ketosteroid Isomerase. Science 346, 1510–1514. 10.1126/science.1259802 25525245 PMC4668018

[B19] Gonçales V. R. Lian J. Gautam S. Tilley R. D. Gooding J. J. (2020). Functionalized Silicon Electrodes in Electrochemistry. Annu. Rev. Anal. Chem. 13, 135–158. 10.1146/annurev-anchem-091619-092506 32289237

[B20] Gooding J. J. Ciampi S. (2011). The Molecular Level Modification of Surfaces: from Self-Assembled Monolayers to Complex Molecular Assemblies. Chem. Soc. Rev. 40, 2704–2718. 10.1039/c0cs00139b 21290036

[B21] Gryn'ova G. Marshall D. L. Blanksby S. J. Coote M. L. (2013). Switching Radical Stability by pH-Induced Orbital Conversion. Nat. Chem 5, 474–481. 10.1038/nchem.1625 23695628

[B22] Gryn’ova G. Coote M. L. (2013). Origin and Scope of Long-Range Stabilizing Interactions and Associated SOMO–HOMO Conversion in Distonic Radical Anions. J. Am. Chem. Soc. 135, 15392–15403. 24090128 10.1021/ja404279f

[B23] Hammill C. L. Noble B. B. Norcott P. L. Ciampi S. Coote M. L. (2019). Effect of Chemical Structure on the Electrochemical Cleavage of Alkoxyamines. J. Phys. Chem. C 123, 5273–5281. 10.1021/acs.jpcc.8b12545

[B24] Higashi G. S. Chabal Y. J. Trucks G. W. Raghavachari K. (1990). Ideal Hydrogen Termination of the Si (111) Surface. Appl. Phys. Lett. 56, 656–658. 10.1063/1.102728

[B25] Hill C. D. Peretz E. Hile S. J. House M. G. Fuechsle M. Rogge S. (2015). A Surface Code Quantum Computer in Silicon. Sci. Adv. 1, e1500707. 10.1126/sciadv.1500707 26601310 PMC4646824

[B26] Hong W. Li H. Liu S.-X. Fu Y. Li J. Kaliginedi V. (2012). Trimethylsilyl-Terminated Oligo(phenylene Ethynylene)s: An Approach to Single-Molecule Junctions with Covalent Au-C σ-Bonds. J. Am. Chem. Soc. 134, 19425–19431. 10.1021/ja307544w 23126569

[B27] Jansen R. (2012). Silicon Spintronics. Nat. Mater 11, 400–408. 10.1038/nmat3293 22522640

[B28] Jaroš A. Bonab E. F. Straka M. Foroutan-Nejad C. (2019). Fullerene-based Switching Molecular Diodes Controlled by Oriented External Electric fields. J. Am. Chem. Soc. 141, 19644–19654. 10.1021/jacs.9b07215 31744293

[B29] Klinska M. Smith L. M. Gryn'ova G. Banwell M. G. Coote M. L. (2015). Experimental Demonstration of pH-dependent Electrostatic Catalysis of Radical Reactions. Chem. Sci. 6, 5623–5627. 10.1039/c5sc01307k 29861899 PMC5949849

[B30] Laviron E. (1974). Surface Linear Potential Sweep Voltammetry. J. Electroanalytical Chem. Interfacial Electrochemistry 52, 395–402. 10.1016/s0022-0728(74)80449-3

[B31] Linford M. R. Chidsey C. E. D. (1993). Alkyl Monolayers Covalently Bonded to Silicon Surfaces. J. Am. Chem. Soc. 115, 12631–12632. 10.1021/ja00079a071

[B32] Linford M. R. Fenter P. Eisenberger P. M. Chidsey C. E. D. (1995). Alkyl Monolayers on Silicon Prepared from 1-alkenes and Hydrogen-Terminated Silicon. J. Am. Chem. Soc. 117, 3145–3155. 10.1021/ja00116a019

[B33] Meir R. Chen H. Lai W. Shaik S. (2010). Oriented Electric Fields Accelerate Dielsâ€"Alder Reactions and Control theendo/exoSelectivity. ChemPhysChem 11, 301–310. 10.1002/cphc.200900848 19998402

[B34] Meldal M. Tornøe C. W. (2008). Cu-Catalyzed Azide−Alkyne Cycloaddition. Chem. Rev. 108, 2952–3015. 10.1021/cr0783479 18698735

[B35] Niermann N. Degefa T. H. Walder L. Zielke V. Steinhoff H.-J. Onsgaard J. (2006). Galvinoxyl Monolayers on Au(111) Studied by STM, EPR, and Cyclic Voltammetry. Phys. Rev. B 74, 235424. 10.1103/physrevb.74.235424

[B36] Niwano M. Takeda Y. Ishibashi Y. Kurita K. Miyamoto N. (1992). Morphology of Hydrofluoric Acid and Ammonium Fluoride‐treated Silicon Surfaces Studied by Surface Infrared Spectroscopy. J. Appl. Phys. 71, 5646–5649. 10.1063/1.350497

[B37] Noble B. B. Norcott P. L. Hammill C. L. Ciampi S. Coote M. L. (2019). Mechanism of Oxidative Alkoxyamine Cleavage: the Surprising Role of the Solvent and Supporting Electrolyte. J. Phys. Chem. C 123, 10300–10305. 10.1021/acs.jpcc.9b01832

[B38] Norcott P. L. Hammill C. L. Noble B. B. Robertson J. C. Olding A. Bissember A. C. (2019). TEMPO-me: An Electrochemically Activated Methylating Agent. J. Am. Chem. Soc. 141, 15450–15455. 10.1021/jacs.9b08634 31483627

[B39] Peiris C. R. Vogel Y. B. Le Brun A. P. Aragonès A. C. Coote M. L. Díez-Pérez I. (2019). Metal-Single-Molecule-Semiconductor Junctions Formed by a Radical Reaction Bridging Gold and Silicon Electrodes. J. Am. Chem. Soc. 141, 14788–14797. 10.1021/jacs.9b07125 31455076

[B40] Rajeshwar K. (2007). Fundamentals of Semiconductor Electrochemistry and Photoelectrochemistry. Encyclopedia of Electrochemistry 6, 1–53.

[B41] Rakshit T. Liang G.-C. Ghosh A. W. Datta S. (2004). Silicon-based Molecular Electronics. Nano Lett. 4, 1803–1807. 10.1021/nl049436t

[B42] Shaik S. Mandal D. Ramanan R. (2016). Oriented Electric fields as Future Smart Reagents in Chemistry. Nat. Chem 8, 1091–1098. 10.1038/nchem.2651 27874869

[B43] Shaik S. Ramanan R. Danovich D. Mandal D. (2018). Structure and Reactivity/selectivity Control by Oriented-External Electric fields. Chem. Soc. Rev. 47, 5125–5145. 10.1039/c8cs00354h 29979456

[B44] Starr R. L. Fu T. Doud E. A. Stone I. Roy X. Venkataraman L. (2020). Gold-Carbon Contacts from Oxidative Addition of Aryl Iodides. J. Am. Chem. Soc. 142, 7128–7133. 10.1021/jacs.0c01466 32212683

[B45] Stewart M. P. Buriak J. M. (1998). Photopatterned Hydrosilylation on Porous Silicon. Angew. Chem. Int. Edition 37, 3257–3260. 10.1002/(sici)1521-3773(19981217)37:23<3257::aid-anie3257>3.0.co;2-1 29711412

[B46] Terry J. Linford M. R. Wigren C. Cao R. Pianetta P. Chidsey C. E. D. (1997). Determination of the Bonding of Alkyl Monolayers to the Si(111) Surface Using Chemical-Shift, Scanned-Energy Photoelectron Diffraction. Appl. Phys. Lett. 71, 1056–1058. 10.1063/1.119726

[B47] Tosi G. Mohiyaddin F. A. Schmitt V. Tenberg S. Rahman R. Klimeck G. (2017). Silicon Quantum Processor with Robust Long-Distance Qubit Couplings. Nat. Commun. 8, 450–460. 10.1038/s41467-017-00378-x 28878207 PMC5587611

[B48] Vilan A. Cahen D. (2017). Chemical Modification of Semiconductor Surfaces for Molecular Electronics. Chem. Rev. 117, 4624–4666. 10.1021/acs.chemrev.6b00746 28230354

[B49] Vogel Y. B. Molina A. Gonzalez J. Ciampi S. (2019). Quantitative Analysis of Cyclic Voltammetry of Redox Monolayers Adsorbed on Semiconductors: Isolating Electrode Kinetics, Lateral Interactions, and Diode Currents. Anal. Chem. 91, 5929–5937. 10.1021/acs.analchem.9b00336 30938142

[B50] Vogel Y. B. Zhang L. Darwish N. Gonçales V. R. Le Brun A. Gooding J. J. (2017). Reproducible Flaws Unveil Electrostatic Aspects of Semiconductor Electrochemistry. Nat. Commun. 8, 2066–2074. 10.1038/s41467-017-02091-1 29233986 PMC5727234

[B51] Wang C. Danovich D. Chen H. Shaik S. (2019). Oriented External Electric fields: Tweezers and Catalysts for Reactivity in Halogen-Bond Complexes. J. Am. Chem. Soc. 141, 7122–7136. 10.1021/jacs.9b02174 30945542

[B52] Warshel A. Sharma P. K. Kato M. Xiang Y. Liu H. Olsson M. H. M. (2006). Electrostatic Basis for Enzyme Catalysis. Chem. Rev. 106, 3210–3235. 10.1021/cr0503106 16895325

[B53] Xiang L. Tao N. J. (2016). Reactions Triggered Electrically. Nature 531, 38–39. 10.1038/531038a 26935691

[B54] Zang Y. Zou Q. Fu T. Ng F. Fowler B. Yang J. (2019). Directing Isomerization Reactions of Cumulenes with Electric fields. Nat. Commun. 10, 4482–4488. 10.1038/s41467-019-12487-w 31578333 PMC6775130

[B55] Zhang L. Ciampi S. Gooding J. J. (2020). Electrostatic Regulation of TEMPO Oxidation by Distal Molecular Charges. ChemElectroChem 7, 3522–3527. 10.1002/celc.202000817

[B56] Zhang L. Espíndola R. B. D. Noble B. B. Gonçales V. R. Wallace G. G. Darwish N. (2018b). Switchable Interfaces: Redox Monolayers on Si(100) by Electrochemical Trapping of Alcohol Nucleophiles. Surfaces 1, 3–11. 10.3390/surfaces1010002

[B57] Zhang L. Laborda E. Darwish N. Noble B. B. Tyrell J. H. Pluczyk S. (2018a). Electrochemical and Electrostatic Cleavage of Alkoxyamines. J. Am. Chem. Soc. 140, 766–774. 10.1021/jacs.7b11628 29258306

[B58] Zhang L. Vogel Y. B. Noble B. B. Gonçales V. R. Darwish N. Brun A. L. (2016). TEMPO Monolayers on Si(100) Electrodes: Electrostatic Effects by the Electrolyte and Semiconductor Space-Charge on the Electroactivity of a Persistent Radical. J. Am. Chem. Soc. 138, 9611–9619. 10.1021/jacs.6b04788 27373457

[B59] Zhang X. G. (2007). Electrochemistry of Silicon and its Oxide: Springer Science & Business Media.

